# Provoking or backfiring? A contingent model of how abusive supervision influences learning from failure through fear

**DOI:** 10.3389/fpsyg.2026.1532064

**Published:** 2026-04-22

**Authors:** Haizhen Wang, Ge An, Jiajia Xu, Lin Ding, Jie Chen

**Affiliations:** 1 School of Business, Xi’an International Studies University, Xi'an, China; 2 School of Business, Nanjing University, Nanjing, China

**Keywords:** abusive supervision, double-edge effects, fear of failure, learning from failure, task variety

## Abstract

**Introduction:**

Unlike prior research that has primarily examined the dysfunctional—and occasionally functional—effects of abusive supervision in general work settings, this study focuses specifically on the context of failure. It investigates when fear of failure induced by abusive supervision facilitates learning from failure, and when it stifles such learning. Grounded in conservation of resources theory and job demands-resources theory, we propose that abusive supervision elicits fear of failure in employees, which subsequently impairs their ability to learn from failure in jobs with high task variety, yet may enhance it in jobs with low task variety.

**Methods:**

To test the proposed theoretical framework, we employed a quantitative research design using three-wave survey data. The sample consisted of 189 employees who provided longitudinal responses to minimize common method bias. We measured abusive supervision, fear of failure, task variety, and learning from failure using established academic scales.

**Results:**

The findings demonstrate that abusive supervision is positively associated with fear of failure. Furthermore, task variety moderates not only the relationship between fear of failure and learning from failure, but also the indirect effect of abusive supervision on learning from failure via fear of failure.

**Discussion:**

By extending abusive supervision research from routine performance to failure learning—a context in which employees are especially prone to defensive withdrawal—this study identifies fear of failure as the affective mechanism that captures the fundamental “arousal– depletion” tension inherent in abusive supervision. We further demonstrate that task variety functions as a directional switch, capable of either amplifying the resource drain caused by abuse or buffering its negative effects, even converting fear-driven responses into positive learning outcomes.

## 1 Introduction

Learning from failure is a resource-intensive adaptive process essential for updating individual knowledge and preventing future errors (Sitkin, 1992). However, setbacks often trigger an ego-threat that drives employees into defensive avoidance (Shepherd, 2003; Cannon and Edmondson, 2001). This raises a critical management dilemma: can the “sharp sting” of leaders, such as abusive supervision, provide a corrective stimulus to rupture this barrier? While traditional views emphasize its destructive impact (Tepper, 2007), arguing that a core pathway through which abusive supervision exerts its negative influence is the elicitation of pervasive negative affect (Tepper, 2007; Martinko et al., 2013), the functional perspective of negative emotions suggests negative emotions such as fear may operate as a double-edged sword (George and Zhou, 2002), acting either as a paralyzing cognitive drain or a catalytic wake-up call. Thus, we formulate our core research question (RQ):

*RQ*: Under what conditions does fear induced by abusive supervision facilitate learning from failure, and under what conditions does it stifle such learning?

To understand the dual nature of this affective reaction, we draw on the conservation of resources (COR) theory (Hobfoll, 1989). From this perspective, failure is not merely a performance deficit but a state of profound resource depletion, where an individual’s self-efficacy and psychological energy are significantly compromised (Shepherd, 2003). In such a vulnerable state, abusive supervision acts as a secondary stressor that amplifies these losses, transforming generalized frustration into a deep-seated fear of failure. Traditionally, COR theory suggests that individuals in a resource-depleted state tend to adopt a defensive posture to prevent further loss, which explains why fear often leads to cognitive paralysis and withdrawal (Tepper, 2007). However, the theory also posits a preventative investment logic: when faced with the threat of continuous resource erosion-specifically, ongoing hostility from a supervisor – employees may be compelled to invest their remaining resources into the arduous task of learning from failure. By doing so, they seek to rectify their performance and terminate the source of the threat, effectively using learning as a survival mechanism to regain environmental control.

Crucially, the outcome of this affective tension – whether it leads to cognitive paralysis or functional adaptation in the observed task-hinges on the resource-demanding nature of the task. By integrating the JD-R model with COR theory, we identify task variety as the pivotal boundary condition that determines the cognitive price of learning. In high-variety roles, learning is an exorbitant endeavor that requires vast resources to navigate complex causal chains; here, the fear induced by abuse creates a resource chokepoint that triggers defensive paralysis. Conversely, in low-variety roles, the structural simplicity reduces these cognitive demands, acting as a resource buffer that neutralizes the toxicity of abuse. This setting can even channel fear into a motivational force that promotes learning. By synthesizing these frameworks, we clarify how task variety determines whether leader hostility results in a devastating resource drain or remains relatively benign.

By investigating these relationships, our research makes three contributions. First, we extend the scope of abusive supervision research from routine work performance to the critical developmental domain of failure learning, a context where employees are particularly prone to defensive withdrawal. Second, we identify fear of failure as a novel affective mechanism that captures the primordial “arousal-depletion” tension of abuse, distinguishing our findings from existing rational-cognitive and motive-based explanations. Third, we shift the focus of contingent factors from individual differences to structural job characteristics, demonstrating that task variety acts as a directional switch that either amplifies the drain of abuse or serves as a structural buffer neutralizing its negative effect and even turning it into a positive. In practice, these insights enable organizations to adopt a precision management approach, tailoring leadership interventions to the specific cognitive demands of different job roles to prevent resource overload and maximize learning potential.

## 2 Literature review

### 2.1 The emergence of double-edged effects of abusive supervision

Abusive supervision, defined as a supervisor’s sustained display of hostile verbal and nonverbal behaviors, excluding physical contact (Tepper, 2000, p. 178), has traditionally been conceptualized as a purely destructive force that depletes subordinate resources and triggers counterproductive work behaviors (Tepper et al., 2017). However, recent scholarly discourse has shifted toward a more nuanced, double-edged perspective, acknowledging that supervisor hostility does not always result in impairment (Fischer et al., 2021). Abusive supervision has been found to yield functional outcomes such as helping (Tröster and Van Quaquebeke, 2021), organizational citizenship behaviors (Yu and Duffy, 2021), and enhanced performance (Liao et al., 2021; Wang et al., 2024) under specific circumstances. This paradigm shift highlights the complexity of subordinate responses. Yet, the current literature primarily observes these double-edged effects within general performance contexts. There is a notable lack of research addressing these dynamics in high-stakes deadlock situations, such as learning from failure. Since work failure often triggers defensive avoidance and psychological inertia (Shepherd, 2003), it represents a critical boundary where the “sharp sting” of abuse might serve a unique, necessary function in rupturing such inertia—a role that the existing double-edged framework has yet to substantiate.

### 2.2 Theoretical explanations of double-edged effects

To explain why abusive supervision yields divergent outcomes, existing research has primarily relied on rational-cognitive and trait-based mechanisms. The most prominent explanation is rooted in attribution theory, which posits that a subordinate’s interpretation of the leader’s intent determines the behavioral path. When abuse is attributed to performance promotion motives, it leads to constructive adaptations like reflectivity or experience self-conscious emotions like guilt, both of which prompt improvement (Tröster and Van Quaquebeke, 2021). Conversely, when the abuse is attributed to injury initiation motives, it triggers dysfunctional reactions such as cognitive rumination (Liao et al., 2021) and destructive emotions like anger, which ultimately erode performance and increase counterproductive behaviors (Yu and Duffy, 2021). Similarly, achievement goal theory suggests that subordinates’ responses are filtered through their internal motivations. For instance, those with a performance-approach orientation may view abuse as a signal to prove competence (Jiang and Lin, 2025; Wang et al., 2024), whereas those with an avoidance orientation perceive it as a threat, leading to performance withdrawal (Wang et al., 2024).

While these theories successfully explain the divergence in behaviors, they largely frame the double-edged effect as a result of deliberate cognitive processing or stable personality filters. This leaves a significant gap in the literature regarding the primordial, pain-driven affective dynamics of abuse. According to COR theory, negative stimuli like abuse elicit intense emotions such as fear of failure, which inherently possesses a double-edged quality: it acts as a high-arousal catalyst to stop further resource loss, yet simultaneously serves as an affective strain that drains cognitive capacity. Understanding this purely affective arousal-depletion tension is vital for explaining how the “sharp sting: compels behavior in contexts where rationalizations may be secondary to the immediate experience of pain.

### 2.3 Contingent factors of the double-edged effects: individual traits

Regarding the conditions under which abusive supervision yields divergent outcomes, the literature has extensively cataloged various individual differences as moderators that steer subordinates toward either constructive or destructive paths. In the trait-based perspective, research has established that subordinates’ goal orientations are decisive in determining the direction of the effect. For instance, while high performance-approach goal orientation enables subordinates to channel the “sting” of abuse into increased work effort to prove their competence, high performance-avoidance goal orientation exacerbates the threat, leading to significant performance withdrawal (Wang et al., 2024). Besides, attribution styles and tendencies play a decisive role in filtering the double-edged impact. Individuals with a performance promotion attribution tendency are more likely to interpret supervisor hostility as a functional (albeit painful) prompt for improvement, which leads to functional adaptation. In contrast, those with an injury initiation attribution tendency perceive the abuse as a malicious attack, which triggers dysfunctional responses (Liao et al., 2021; Tröster and Van Quaquebeke, 2021; Yu and Duffy, 2021). Additionally, other dispositional factors – such as an internal locus of control, dispositional forgiveness, and strong ethical values (e.g., Islamic work ethic) – have been identified as critical diverters that steer subordinates away from retaliation and toward self-improvement (Kim et al., 2020; Islam et al., 2021).

Collectively, these studies provide a comprehensive map of *who* (based on motivational and attributional traits) is likely to navigate the double-edged sword of supervisor hostility. However, this focus on subjective willingness and cognitive framing largely neglects the objective structural constraints of the task itself. Even when a subordinate possesses the correct attribution style (e.g., promotion-focused) or high motivation, the feasibility of a functional response remains bounded by the resources required to enact it. In contexts like learning from failure, the task variety defines the “cognitive price tag” of the response. Without accounting for these structural boundaries, the literature remains unable to explain why even well-intended or resilient subordinates may fail to translate the “sharp sting” into learning when the task’s resource demands exceed their threshold. This suggests that the double-edged effect is not only a matter of perception but is strictly constrained by the structural affordability of the task.

To address these gaps, this study develops a moderated mediation model grounded in the conservation of resources theory and the job demands-resources model. Specifically, it proposes that abusive supervision increases employees’ fear of failure. Furthermore, the study examines task variety as a key moderator, suggesting that the fear of failure inhibits learning from failure when task variety is high, whereas it facilitates learning from failure when task variety is low. By investigating the interplay between abusive supervision, fear of failure, and task variety, this research aims to provide a more nuanced understanding of the double-edged influence of abusive supervision in the context of work failure.

## 3 Theoretical background and hypotheses development

### 3.1 Conservation of resource theory

Conservation of resource (COR) theory serves as the primary framework for understanding how individuals manage their personal and energetic resources under stress. The theory is structured around several fundamental principles regarding resource loss, investment, and the strategic allocation of remaining reserves. A core tenet of COR theory is the primacy of resource loss, which posits that resource loss is disproportionately more salient than resource gain (Hobfoll, 1989; Hobfoll et al., 2018). Individuals have a fundamental drive to obtain, retain, and protect their resources – defined as objects, personal characteristics, conditions, or energies that are valued (Hobfoll, 2001). Because loss is perceived as more impactful than an equivalent gain, any signal of potential resource depletion triggers an intense psychological state of threat. This aversion to loss functions as a primary survival mechanism; when individuals sense that their current resource pool is being compromised, they experience heightened affective states that serve as an alarm system, signaling the urgent need for protective action to prevent further exhaustion (Halbesleben et al., 2014).

Furthermore, COR theory describes two distinct behavioral trajectories in response to such threats. The first is preventative investment, where individuals strategically deploy remaining resources to forestall further loss (Hobfoll, 2001). The second is the loss spiral, occurring when the resources required to cope with an initial stressor leave the individual with insufficient capacity for subsequent demands (Hobfoll, 1989). When individuals perceive no clear path to recovery, they often resort to defensive resource conservation, pulling back effort and detaching from the stressor to prevent total exhaustion (Halbesleben et al., 2014).

The COR theory also clarifies that resource-depleted or threatened individuals do not allocate their remaining energy randomly; rather, they carefully select the manner in which they use their remaining resources to maximize survival (Siegall and McDonald, 2004). Individuals will invest resources as a coping mechanism only where the greatest potential return on investment is possible (Freedy and Hobfoll, 1994). This resource calculus suggests that behaviors perceived as highly instrumental for stopping resource loss – such as meeting formal performance expectations – are prioritized over discretionary or extra-role behaviors (Organ, 1988). Consequently, the decision to engage in resource-intensive functional behaviors (like learning from failure) is governed by the affordability of the response (Halbesleben et al., 2014). If the environmental demands are excessive, the cost of investment becomes prohibitive, forcing individuals to abandon proactive strategies in favor of withdrawal to protect their remaining reservoir (Hobfoll and Shirom, 2001). In the context of abusive supervision, researchers have utilized COR theory as a primary lens (Tu and Chi, 2024; Rus et al., 2025) because it moves beyond mere affective reactions to capture the systematic depletion of resources.

### 3.2 Job demand-resource model

The Job demand-resource (JD-R) model provides a structural framework to evaluate the work environment through two fundamental categories: job demands and job resources (Bakker and Demerouti, 2007; Demerouti et al., 2001). Job demands encompass the physical, social, or organizational factors at work that necessitate sustained physical or psychological effort. Conversely, job resources are the work factors that aid workers in achieving their goals and mitigate the demands and associated physical and mental strain. The JD-R model posits that while resources initiate a motivational process, excessive demands trigger a health-impairment process that exhausts an individual’s mental energy (Bakker and Demerouti, 2017).

A core extension of the JD-R model is the logic of resource-demand fit, which suggests that employee outcomes are determined by the alignment between the level of available resources and the intensity of job demands (Bakker et al., 2007). While much of the literature emphasizes how resources can buffer demands, the model also underscores a reciprocal interaction: high job demands can interfere with the utility of resources. Specifically, the “Matching Hypothesis” within JD-R suggests that for a functional behavior to occur, the individual must possess specific resources that fit the nature of the demand (De Jonge and Dormann, 2006). If the demands of a task are so high that they exceed the individual’s current resource capacity, a state of mismatch occurs, rendering even high levels of motivation ineffective. This fit perspective establishes that job demands act as a structural gatekeeper for the motivational process. According to the JD-R model, the positive effect of a motivational state on performance is only sustained when the environment provides a resource margin – a surplus of energy where resources comfortably outweigh demands. When the environment imposes a high “price of entry” (high demands), it creates a bottleneck that prevents the translation of motivation into action. In such cases, the demand does not just coexist with the resource; it actively constrains the resource’s functional potential. This provides a theoretical basis for why an individual, even when driven by a potent internal state, may fail to engage in resource-intensive behaviors if the structural fit between their remaining energy and the task’s complexity is absent. Therefore, recent studies on destructive leadership have frequently paired JD-R with COR to map how environmental demands interact with internal resource states (Bakker and Demerouti, 2017; Malik et al., 2023).

### 3.3 Theoretical integration

The integration of COR theory and the JD-R model provides a holistic framework for explaining the double-edged outcomes of abusive supervision. This study posits that the “sharp sting” of supervisor hostility functions as a high-stakes resource catalyst, where the final behavioral outcome is determined by a dynamic interplay between psychological drive and environmental affordability.

While COR theory explains the internal motivational divergence, it establishes that the threat of resource loss triggers an intense affective state (fear of failure) that forces a strategic choice: subordinates may either engage in preventative investment (learning) to forestall further loss, or succumb to a loss spiral (withdrawal of learning) to conserve remaining energy. However, COR primarily addresses the *intent* to navigate this double-edged sword. To provide a complete picture, the JD-R model introduces the structural constraints that dictate which side of the sword will strike. By framing task characteristics as job demands that consume the same finite resource pool required for investment, the JD-R model defines the resource-demand fit.

Together, these theories establish a dual-path logic: the “sharp sting” creates the *tension* necessary for either functional or dysfunctional change (COR), but the task environment determines the *feasibility* of the functional path (JD-R). Abusive supervision only yields a benefit when the task’s resource demands are low enough to allow for a successful resource-demand fit. When the task is too demanding, the structural bottleneck forces the “sting” into a purely destructive trajectory. Thus, the integrated framework explains not only *why* the double-edged effect exists but *when* the functional edge remains sharp versus when it collapses into total impairment.

## 4 Hypothesis development

### 4.1 Abusive supervision and fear of failure

Abusive supervision, characterized by the sustained display of hostile verbal and non-verbal behaviors, constitutes a severe and ongoing threat to a subordinate’s resource reservoir (Tepper, 2000). According to COR theory, individuals are fundamentally motivated to protect their existing resources and are hyper-sensitive to the threat of further depletion (Hobfoll, 1989; Hobfoll et al., 2018). Supervisor abuse directly jeopardizes several valued resources, including career development, organizational status, and social support. As supervisors are primary gatekeepers of workplace rewards – such as raises, promotions, and continued employment (Xu et al., 2015) – their hostility signifies not only a disapproval of the employee’s contributions but a tangible threat to the employee’s future professional security. Furthermore, because supervisors serve as critical sources of role clarification and workplace support (Halbesleben, 2006; Jokisaari and Nurmi, 2009), their abuse signals the sudden withdrawal of social resources, leaving employees in a vulnerable and resource-depleted state (Aryee et al., 2008).

The primacy of resource loss tenet of COR theory suggests that when individuals face such significant resource threats, they develop an intense aversion to any situation that might trigger additional loss (Hobfoll et al., 2018; Kiewitz et al., 2016). In this context, work failure – defined as outcomes that fall short of performance expectations (Dahlin et al., 2018) – is no longer perceived as a mere setback but as a high-risk catalyst for further resource erosion. We argue that abusive supervision heightens the fear of failure through two parallel mechanisms of threat.

First, failure carries a profound social stigma in an abusive context. It signifies poor performance that can lead supervisors to perceive the employee as harmful or threatening to the unit’s goals and efficiency. According to the logic of moral exclusionary practices, this negative perception allows the supervisor to feel psychologically justified in escalating hostile behaviors, viewing abuse not as a transgression but as a form of social punishment for the employee’s perceived lack of utility (Tepper et al., 2011). Second, work failure significantly increases an employee’s negative visibility, subjecting them to closer and more critical scrutiny. Because failure reveals a clear and quantifiable discrepancy between aspirations and actual performance (Wilhelm et al., 2019), it provides focal point for the supervisor’s aggressive attention. For a subordinate already suffering from resource erosion, this heightened visibility transforms the workplace into panopticon where any subsequent minor slip-up is amplified, making the employee a more frequent and vulnerable target for future abuse.

Consequently, for employees already suffering from resource erosion due to prior abuse, these parallel threats – stigmatization and scrutiny – transform the prospect of failure into an imminent danger to their remaining resource pool. This intense aversion culminates in a state of fear of failure—an appraisal-to-threat concept where individuals perceive evaluative situations as dangerous to their self-preservation (Conroy, 2004). Previous research supports this link, noting that an individual’s social environment, particularly the behavior of leaders, is a primary determinant of failure-related anxiety (Dias and Martens, 2019; Huang, 2021). Thus, we propose that abusive supervision heightens an employee’s apprehension toward failure as a defensive reaction to protect their remaining resources.

*Hypothesis 1*: Abusive supervision is positively associated with employees’ fear of failure.

### 4.2 The dual-path effects of fear of failure on learning from failure

Learning from failure is a resource-intensive cognitive process involving spontaneous reflection on failure events and behavioral adjustments to prevent future recurrences (Green et al., 2003). Drawing on the COR theory and the JD-R model, we argue that the impact of fear of failure on this learning process is inherently complex. This fear represents a high-arousal affective state that functions simultaneously as a resource drain and a motivational signal, leading to divergent effects on failure learning.

#### 4.2.1 The negative path: resource depletion and cognitive interference

Fear of failure may impede failure learning through two primary mechanisms of resource depletion. First, fear distorts critical resource allocation (Bower, 1992; Fredrickson, 2001). According to COR theory, when individuals perceive a severe threat, their cognitive energy is redirected toward threat monitoring – focusing on the potential consequences of failure (e.g., supervisor hostility) rather than the failure event itself (Meinhardt and Pekrun, 2003). This attentional narrowing reduces the cognitive capacity available for the deep, integrative, and creative thinking required to analyze complex failure causes (Fredrickson and Branigan, 2005; Hirak et al., 2012).

Second, fear erodes the intrinsic motivation necessary for sustainable resource investment. Fear activates negative mental schemas and increases sensitivity to negative stimuli (Lattacher and Wdowiak, 2020), leading to a pessimistic appraisal of one’s ability to find solutions. From the perspective of the health-impairment process in the JD-R model, such prolonged emotional strain exhausts the individual’s energetic reserves, making them less willing to gamble their remaining energy on learning activities that offer uncertain returns (Shepherd et al., 2011). Just as emotional security enables exploration in learning environments, fear triggers a defensive withdrawal, disengaging the individual from the failure-making process to prevent further exhaustion (Bergin and Bergin, 2009; D’Mello and Graesser, 2012).

#### 4.2.2 The positive path: defensive investment and the early warning system

Conversely, fear of failure can function as a potent extrinsic catalyst for failure learning. From an evolutionary perspective, fear serves as an internal early warning system that signals an immediate threat to survival (Leary, 1983; Sweeney and Pine, 2004). Within the COR framework, when the “sharp sting” of abusive supervision makes the cost of future failure catastrophic, the fear of that loss can trigger preventative investment.

In this scenario, subordinates may perceive failure learning as the most effective defense strategy to minimize their negative visibility and stop the ongoing erosion of resources (Byrne and Shepherd, 2015). By identifying root causes and developing solutions, employees seek to repair their performance standing to appease the supervisor and avoid further victimization. This suggests that negative emotions do not always obstruct sense-making; rather, when the pressure is sufficiently high, they can motivate intense, focused reflection as a means of survival (Dias and Teixeira, 2017).

#### 4.2.3 Theoretical synthesis: a competing influence perspective

Based on the arguments above, we posit that fear of failure exerts competing influences on learning from failure. On one hand, it acts as a cognitive tax that impairs the capacity to learn (the impairment path); on the other hand, it acts as a motivational fuel that provides the impetus to learn (the investment path). Because these two paths – depletion and motivation – operate simultaneously, the net effect of fear of failure on failure learning may depend on the specific resource-demand configuration of the environment.

Given these theoretically sound but opposing mechanisms, the overall direction of the relationship between fear of failure and failure learning is complex and may be subject to significant variance. Similarly, the resulting indirect effect of abusive supervision on learning from failure via fear of failure remains theoretically ambiguous. Therefore, in line with the dual-path logic of COR and JD-R, we treat these relationships as competing processes and refrain from hypothesizing a specific unidirectional mediation effect, focusing instead on identifying the boundary conditions that determine which path prevails.

### 4.3 The moderating role of task variety

Building upon the dual-path logic established in Section 4.2, we propose that task variety functions as the critical determinant of which path prevails. Task variety acts as a structural gatekeeper that dictates the resource affordability of failure learning, thereby toggling the valence of the relationship between fear of failure and learning outcomes.

Task variety refers to the degree to which a job requires an employee to perform a wide range of diverse tasks (Morgeson and Humphrey, 2006). In high-variety roles, failure events are often multifaceted and embedded within complex work processes. Consequently, learning from such failures is exceptionally resource-demanding and difficult; it requires the employee to engage in cross-domain reflection, switch between different cognitive schemas, and synthesize disparate information to identify root causes (Monsell, 2003). These inherent characteristics suggest that the resource for successful learning in high-variety contexts is significantly higher, but the probability of successful learning in high-variety contexts is significantly lower than in low-variety tasks, where failures are typically repetitive, and their solutions are more straightforward (Pan et al., 2018).

Thus, we argue that the high-resource nature of these tasks makes the learning process hyper-sensitive to the resource depletion caused by the fear of failure. As aforementioned, fear of failure – triggered by the “sharp sting” of abuse – functions as a primary drain on an employee’s resource reservoir. When task variety is high, the “resource price” of learning often exceeds the employee’s remaining supply. Even if the abused employee is motivated to learn as preventative investment to avoid future victimization, they simply cannot afford the resource cost required by the complex task. Furthermore, the structural complexity of high-variety tasks and the difficulty of learning from prior failures in this type of task reinforce the pessimism inherent in the fear of failure (Section 4.2.1). Subordinates may perceive that the likelihood of a successful fix is slim and that effort may be futile. Faced with a high-cost, low-probability investment, the employee abandons the investment path and shifts to a defensive posture, reducing learning behaviors to protect their remaining resources from further wasteful expenditure (Hobfoll et al., 2018).

Conversely, in low-variety contexts, the resource price of learning is low, and the path to success is clear. This task feature buffers the employee against the negative impact of fear: the resource tax from fear does not deplete the employee below the threshold required for simple learning, and the high probability of success counteracts fear-induced pessimism. In such cases, the defensive motivator of fear can be successfully channeled into learning.

*Hypothesis 2*: Task variety moderates the relationship between fear of failure and employee learning from failure, such that for employees engaged in high-variety tasks, fear of failure is negatively related to learning from failure, whereas for employees engaged in low-variety tasks, fear of failure is positively related to learning from failure.

### 4.4 The integrative moderated mediation model

Building upon the integration of COR and JD-R theories, we propose a moderated mediation model to explain the contingent nature of the double-edged effect. This hypothesis posits that the ultimate behavioral outcome of the fear of failure, triggered by abusive supervision, is dictated by the structural demands of the task environment.

As proposed in Hypothesis 1, hostile behaviors from supervisors erode an employee’s professional resources and psychological safety, transforming work failure into an existential threat. This induces a state of fear of failure—an affective response that is inherently neutral in its potential; it contains both the motivational potential to avoid further loss and the cognitive load that threatens to deplete existing resources.

Based on the logic of Hypothesis 2, whether the fear sparked by abuse translates into constructive learning or a destructive collapse depends entirely on the task configuration. Under high task variety, the indirect path manifests as a negative depletion chain. Because learning in high-variety tasks has an exceptionally high entry price, the cognitive resources already taxed by fear become insufficient. This resource-demand mismatch, combined with low expectations of success in a complex environment, collapses the employee’s willingness to invest. Consequently, abusive supervision exerts a significant negative indirect effect on failure learning via fear of failure. Under low task variety, the indirect path transforms into a positive motivational chain. Because the learning requirements are straightforward and low-cost, the fear of failure functions as a defensive motivator. Subordinates can successfully channel their apprehension into focused reflection without being overwhelmed by task complexity. Thus, abusive supervision exerts a significant positive indirect effect on failure learning via fear of failure.

**Figure 1:**
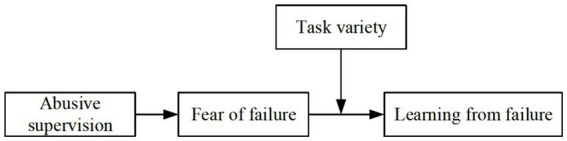
Conceptual framework.

In summary, abusive supervision does not lead to a uniform outcome of withdrawal or growth. Its influence, transmitted through the fear of failure, is governed by resource-demand fit – the alignment between the employee’s remaining resource supply and the diverse demands of their tasks. Recent empirical research lends support to the idea that job characteristics can modulate the effects of stressors such as abusive supervision. For example, Wu et al. (2018) found that the detrimental impact of destructive leadership on role stress was amplified under conditions of high job complexity, leading to increased employee silence. Furthermore, studies have shown that high job demands can exacerbate the dysfunctional effects of destructive leadership. Kunz and Millhoff (2023), for instance, reported that high job demands aggravated the negative impact of destructive leadership on employee work ability. Taken together, these findings underscore the importance of considering job characteristics, including job demands, as contextual contingencies that shape the effects of destructive leadership on employee outcomes. Our conceptual framework is illustrated in Figure 1.

*Hypothesis 3*: Task variety moderates the indirect effect of abusive supervision on learning from failure via fear of failure. Specifically, the indirect effect is negative when task variety is high, and positive when task variety is low.

## 5 Methods

### 5.1 Data and sample

We administered surveys at three different time points with a two-week interval between each. At Time 1, participants rated their demographic characteristics and task variety. At Time 2, employees reported abusive supervision and fear of failure. At Time 3, employees rated their learning from failure. We collected data at the employee level because the fear of failure and the psychological mechanisms of the learning process occur at the individual level. To encourage participation, each respondent received RMB 30 for completing the survey. Using a convenience sampling method, we initially approached 401 participants at Time 1, 392 at Time 2, and 223 at Time 3, resulting in 189 matched responses (a response rate of 47%). Data from three participants were excluded because they self-identified as students and were not currently employed. The adequacy of the sample size was validated through power analysis using G*Power. Given the total sample size of *N* = 189 and 9 predictors in the regression model, the study achieved a power of 0.98 to detect a medium effect size (ƒ^2^ = 0.15) at a significance level of alpha = 0.05, exceeding the commonly accepted threshold of 0.80. Furthermore, a post-hoc power analysis specifically targeting the observed moderation effect (*ΔR*^2^ = 0.06) yielded a power greater than 0.95. These results collectively indicate that the study was sufficiently powered to detect the interaction effect, ensuring the statistical robustness of the findings.

Participants were recruited via WeChat, the most widely used social media platform in China. We initially posted recruitment advertisements within our professional networks and encouraged contacts to forward the advertisement to their respective professional circles. Interested individuals were invited to join dedicated WeChat groups managed by the research team. This human-centrism recruitment approach was intentional: it established a foundation of interpersonal trust, which is crucial for obtaining honest responses regarding sensitive topics like supervisor abuse. To ensure accurate longitudinal matching, we utilized a customized identification code (a combination of letters and digits) created by the respondents, which allowed us to link responses across the three waves while maintaining anonymity. Furthermore, to address response biases and ensure data validity, we embedded attention-check items (e.g., “Please select ‘Highly Disagree’ for this item”) within the survey; only those who passed the attention checks were included, and their responses were pooled for cross-wave matching and analysis.

The respondents are employees from across China, primarily in the Northwest region. The Northwest region of China was selected as the research context because it is currently undergoing rapid industrial transformation. Enterprises in this area face significant competitive pressure and higher failure rates (Zhang and Ma, 2025), making it an ideal setting for investigating how employees learning from failures. They were employed in various industries, including manufacturing, finance, healthcare, information technology, and education. Of the respondents, 61.4% were women (*SD* = 0.49), the majority held an undergraduate degree, and the average age was 33.26 years (*SD* = 7.74). Their average tenure at their current work unit was 5.26 years (*SD* = 5.29), and they had an average of 3.79 (*SD* = 4.17) years working with their current supervisor. The relatively high proportion of female respondents (61.4%) in our study aligns with the industrial composition of the sample, which primarily comprises healthcare, education, and finance. In the Chinese labor market, these specific industries are characterized by a naturally higher concentration of female practitioners (Li et al., 2024; He and Goncalves, 2025). To ensure the empirical rigor of our findings and to partial out any potential confounding effects, we included gender as a control variable. The consistency of our results confirms that the core findings are robust and independent of gender distribution.

### 5.2 Measures

#### 5.2.1 Abusive supervision

Abusive supervision is defined as subordinates’ perceptions of the extent to which supervisors engage in the sustained display of hostile verbal and nonverbal behaviors, excluding physical contact (Tepper, 2000). To measure this construct, Mitchell and Ambrose’s (2007) shortened scale, which includes five items from Tepper’s (2000) 15-item measurement, was adopted. The five-item version is suitable for multi-wave surveys as it captures the core dimensions of the construct while mitigating respondent fatigue (Mitchell and Ambrose, 2007). A sample item is “My supervisor ridicules me”. Using a five-point Likert scale, respondents rated the frequency of each item. Cronbach’s α was 0.95.

#### 5.2.2 Fear of failure

Fear of failure refers to an appraisal-to-threat concept where individuals assess evaluative situations as threatening when there is a potential for failure (Conroy, 2004). We utilized a five-item scale adapted from the Performance Failure Appraisal Inventory (PFAI) developed by Conroy et al. (2002). This scale captures the nature of fear of failure, specifically the loss of social value and the threat to interpersonal resources. One sample item is “When I am failing, I worry about what others think about me.” (1 = strongly disagree, 6 = strongly agree). Cronbach’s α was 0.91.

#### 5.2.3 Task variety

Task variety is defined as the degree to which a job requires an employee to perform a wide range of tasks and involves different skills and talents (Morgeson and Humphrey, 2006). A four-item scale from the Work Design Questionnaire (WDQ) by Morgeson and Humphrey (2006) was used. The WDQ scale is currently recognized as the most comprehensive instrument for measuring work design and is extensively utilized across research fields such as organizational behavior and industrial psychology. Furthermore, its cross-cultural validity has been rigorously established, as it has been translated into multiple languages and widely applied in diverse international contexts (Ríos et al., 2017). A sample item is “My job involves doing a number of different things” (1 = strongly disagree, 6 = strongly agree). Cronbach’s α was 0.93.

#### 5.2.4 Learning from failure

Learning from failure is a cognitive and behavioral process through which individuals analyze errors and negative outcomes to prevent future occurrences and improve performance (Cannon and Edmondson, 2005). We use a six-item scale to measure this process based on the work of Carmeli (2007) and Tucker and Edmondson (2003). This instrument provides a highly accurate and direct measure of the learning-from-failure construct, and it is widely applied in the research of learning from failure (Carmeli et al., 2012). One sample item is “At work, I often ask ‘Is there a better way to produce the product or provide the service?’” (1 = strongly disagree to 6 = strongly agree). Cronbach’s α was 0.90.

#### 5.2.5 Control variables

The previous research indicated that employee’s demographic variables influence their learning behaviors (He et al., 2018; Shepherd et al., 2011). We thus controlled for employees’ gender (1 = female, 0 = male), age, educational background (1 = below junior college degree, 2 = junior college degree; 3 = bachelor’s; 4 = master’s or above). We also controlled for tenure with the leader (in years) due to its potential impact on the individual psychological experience and subsequent learning behavior (Zhou et al., 2020). In line with prior research conducted in the Chinese context (Zhou et al., 2020; Su et al., 2020), we also included firm ownership (1 = state-owned enterprises, others = 0) as a control variable. Employees in state-owned enterprises (SOEs) – often described as holding an “iron rice bowl” – typically enjoy greater job security (Wong, 2018), which can shape their perceptions of leadership pressure and their motivation to learn from failure. Moreover, SOEs are characterized by higher power distance and more rigid hierarchical structures, which may increase the prevalence of destructive supervisory behaviors (Wei and Si, 2013). Controlling for ownership thus helps ensure that the observed relationships among abusive supervision, fear, and learning from failure are not driven by these organizational differences.

### 5.3 Analytical procedures

Data analysis was performed using SPSS 28 and Mplus 8.3 (Muthén and Muthén, 1998–2019) following standard analytical protocols. First, we conducted Harman’s single-factor test and an unmeasured latent method factor analysis to assess potential common method bias. Second, confirmatory factor analyses (CFA) were conducted in Mplus to assess the convergent and discriminant validity of our key constructs. After confirming measurement validity, we computed descriptive statistics and Pearson correlation matrices. For hypothesis testing, we employed hierarchical regression analysis and Hayes (2013) PROCESS macro. Specifically, hierarchical regression was used to test the direct effect (Hypothesis 1) and the moderation effect (Hypothesis 2). Then, the moderated mediation effect (Hypothesis 3) was tested using PROCESS Model 14.

### 5.4 Common method bias

To reduce the possibility of common method variance (CMV) (Podsakoff et al., 2012), data in this study were collected at three different time points with intervals of 2 weeks, a method that has been adopted by studies involving employee learning (Carmeli and Gittell, 2009). As previously mentioned, participants first reported on task variety; 2 weeks later, they rated their experiences of abusive supervision and fear of failure. Two weeks after that, learning from failure was assessed. In addition, Harman’s single-factor test results showed that a single factor accounted for 26% of the total variance, which is lower than the 50% standard, indicating that there was no significant CMV in our data. Furthermore, an unmeasured latent method factor analysis (Williams and McGonagle, 2016) showed that the latent factor explained only 17% of the variance, well below the 25% threshold (Williams et al., 1989). These results collectively indicate that CMB was not a serious concern in this study.

### 5.5 Results

We conducted a series of confirmatory factor analyses (CFAs) using Mplus version 7 to assess the discriminant validity of our measures. The hypothesized four-factor model (abusive supervision, fear of failure, task variety, and learning from failure) provided the best fit for the data (*χ*^2^[164] = 289.12, RMSEA = 0.06, CFI = 0.96, TLI = 0.95), with the chi-square being significantly lower than that of the other models (reported in Supplementary material). Table 1 presents the descriptive statistics and correlations. As expected, abusive supervision was positively correlated with fear of failure (*r* = 0.30, *p* < 0.01). Although years with leader were significantly correlated with age (*r* = 0.57, *p* < 0.01), the variance inflation factors (VIFs < 2) indicated no significant multicollinearity.

**Table 1:** Correlations, means, and standard deviations.

Variable	1	2	3	4	5	6	7	8	9
1. Gender	–								
2. Age	−0.18*	–							
3. Education	0.15*	−0.28**	–						
4. Tenure with the leader	−0.08	0.57**	−0.15*	–					
5. Firm ownership	−0.14*	0.10	0.14	0.10	–				
6. Abusive supervision	−0.04	−0.04	−0.00	−0.02	0.13	**0.95**			
7. Task variety	−0.11	0.03	0.18*	−0.06	0.06	−0.09	**0.93**		
8. Fear of failure	−0.00	−0.09	0.06	−0.11	0.07	0.30**	−0.04	**0.91**	
9. Learning from failure	−0.14*	−0.10	0.14	−0.10	−0.07	−0.03	0.21**	0.01	**0.90**
Means	0.61	33.26	2.92	3.79	0.24	1.79	4.47	3.35	4.44
SD	0.49	7.74	0.68	4.17	0.43	0.87	1.14	1.11	0.75

*N* = 189. Reliabilities (coefficient alphas) are on the diagonal in parentheses. **p < 0.01, *p < 0.05. Bold values on the diagonal in Table represent the Cronbach’s alpha coefficients for each variable.

To test our hypotheses, we performed a series of regression analyses using SPSS 26.0. As shown in Table 2, we first predicted fear of failure by including all control variables (Model 1) and then added abusive supervision (Model 2). We found that abusive supervision was positively associated with fear of failure (*b* = 0.37, *p* < 0.001). Thus, Hypothesis 1, which suggested a positive association between abusive supervision and fear of failure, was supported.

**Table 2:** Results of regression analysis.

Variables	Fear of failure		Learning from failure		
	Model 1	Model 2	Model 3	Model 4	Model 5
Constant	3.45***	2.64***	4.34***	3.97***	1.95**
Gender	−0.02	−0.00	−0.30**	−0.27*	−0.25*
Age	−0.01	−0.00	−0.00	−0.01	−0.00
Education	0.05	0.06	0.18*	0.13	0.11
Tenure with the leader	−0.03	−0.03	−0.01	−0.01	−0.01
Firm ownership	0.21	0.10	−0.19	−0.19	−0.23
Abusive supervision		0.37***		−0.01	−0.03
Fear of failure				0.01	0.63***
Task variety				0.12*	0.55***
Fear of failure × Task variety					−0.13***
*F*	0.77	3.54**	2.63*	2.43*	3.63***
*R* ^2^	0.02	0.10	0.07	0.10	0.15
Δ*R*^2^		0.08***			0.06***

Unstandardized coefficients were reported; ***p < 0 0.001, **p < 0.01, *p < 0.05.

**Figure 2:**
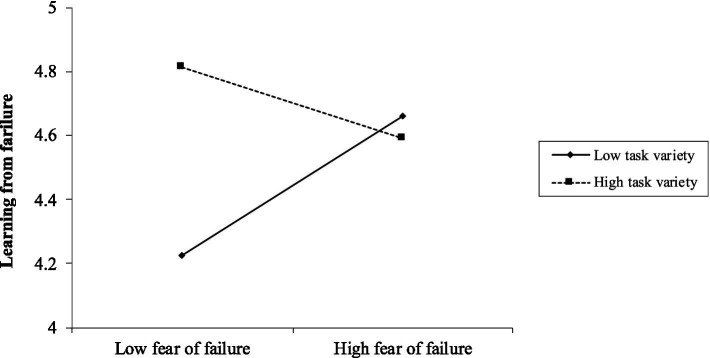
Moderating effect of task variety on fear of failure and learning from failure.

Hypothesis 2 proposed a moderating effect of task variety on the relationship between fear of failure and learning from failure. As shown in Model 5, the interaction term (Fear of Failure × Task Variety) was significantly associated with learning from failure (*b* = −0.13, *p* < 0.001). To elucidate the interaction, we conducted simple slope analyses. The results (see Figure 2) suggest that under low levels of task variety (*M* – 1 SD), the effect of fear of failure on learning from failure was positive and significant (*b* = 0.17, *p* < 0.05); under high levels of task variety (*M* + 1 SD), the effect of fear of failure on learning from failure was negative and significant (*b* = −0.16, *p* < 0.05). Therefore, Hypothesis 2 was supported.

Finally, to examine the moderated mediation effect predicted by Hypothesis 3, we used the PROCSS program (Model 14, Hayes, 2013) to assess the conditional mediating effect of fear of failure using a bootstrapping procedure with 5,000 random samples to replace the full sample. When task variety was low (−1 SD), the size of the indirect effect was 0.06, and the 95% confidence interval, which included zero, was [−0.02, 0.14], suggesting no significant conditional indirect effect. However, at high levels of task variety (+1 SD), the size of the indirect effect was −0.06, and the 95% confidence interval, excluding zero, was [−0.13, −0.00], indicating a significant conditional indirect effect. The index of moderated mediation was −0.05 (95% CI [−0.10, −0.00]), which indicated that the moderated mediation effect was significant, partially supporting Hypothesis 3. Hypothesis 3 posited that task variety moderates the indirect effect of abusive supervision on learning from failure via fear of failure. Specifically, the indirect effect was hypothesized to be negative when task variety is high and positive when it is low. The results supported the first part (high task variety) but not the second (low task variety).

## 6 Discussion

The primary objective of this study was to unravel the double-edged sword effect of abusive supervision on employee learning from failure. By answering how (fear of failure) and when (task variety), this process occurs; our findings provide a nuanced understanding of the functional and dysfunctional outcomes of leader hostility. Consistent with Hypothesis 1, our results established a positive link between abusive supervision and fear of failure. This confirms previous research emphasizing the role of leaders in shaping the workplace emotional climate (e.g., Huang, 2021). In particular, previous research has found that abused employees experience fear, including the fear of negative evaluation (Kiewitz et al., 2016; Selem et al., 2023).

In support of Hypothesis 2, our findings reveal that the relationship between fear of failure and learning is not monolithic. In high-variety contexts, fear of failure is negatively related to learning from failure, whereas in low-variety tasks, fear of failure is positively related to learning from failure. This finding aligns with the dual-path logic proposed by Cheng and McCarthy (2018). They argue that workplace anxiety may lead to debilitative and facilitative job performance under different motivation, ability, and emotional intelligence conditions. Similarly, George and Zhou (2002) identified a double-edged effect where negative affect can either stifle performance through frustration or enhance it by signaling that “things are not going well”, thereby prompting a more detail-oriented and focused analytical style.

Notably, in low-task-variety contexts, our discovery that fear of failure can positively relate to learning presents a meaningful dis-confirmation of the traditional view that fear is universally detrimental. While scholars like D’Mello and Graesser (2012) suggest fear disengages learners, our results support the nuanced perspective of Pekrun (2006) and Byrne and Shepherd (2015) that negative emotions can trigger extrinsic motivation. This implies that when task variety is low, the warning signal of fear might actually push employees to analyze their errors more diligently to avoid future abuse, as the cognitive resources required for such tasks do not exceed the employee’s depleted capacity.

Our results partially support Hypothesis 3. We found that task variety moderates the indirect effect of abusive supervision on learning from failure through fear of failure. Specifically, when task variety is high, abusive supervision indirectly decreases learning from failure through fear of failure. However, when task variety is low, abusive supervision indirectly increases learning from failure through fear of failure, though this positive relationship did not reach statistical significance. This nuanced finding is consistent with the emerging body of research on the functional consequences of abusive supervision, which suggests that the bright side of leader hostility is highly contingent on specific boundary conditions and often manifests with limited intensity. The lack of significance in our positive path (under low task variety) further mirrors the complexity found in existing literature, where functional effects often depend on specific psychological filters or individual traits. Research has shown that abusive supervision leads to constructive outcomes only when subordinates attribute the supervisor’s behavior to performance-promotion motives rather than injury-initiation motives (Liao et al., 2021). Similarly, the positive impact on job performance through work effort is often restricted to individuals with a high performance-approach goal orientation (Wang et al., 2024) or a high achievement orientation (Jiang and Lin, 2025). Some studies suggest that abused subordinates may experience shame and subsequently engage in self-image reparative actions by improving work performance (Liang, 2023). However, this making up process is more likely to occur only among employees with high face threat sensitivity or those who “love face”, driving them to work harder to achieve high performance in the face of abuse (Jiang and Lin, 2025).

### 6.1 Theoretical implications

First, we extend the scope of abusive supervision research from routine work performance to the critical developmental domain of failure learning. Traditionally, research on abusive supervision has focused on general work scenarios, examining its impact on routine task performance, organizational citizenship behavior, or workplace deviance (Tepper, 2000; Tepper et al., 2017). While these outcomes are vital, they often reflect maintenance or compliance behaviors. By shifting the focus to learning from failure, we explore how destructive leadership affects an employee’s capacity for cognitive growth and adaptation following setbacks. This extension is crucial because learning from failure is a high-resource, high-risk activity; our study reveals that leader hostility does more than just lower daily output – it threatens the very mechanisms through which an organization evolves and rectifies errors. By establishing this link, we provide a more comprehensive understanding of the long-term, developmental costs of supervisor hostility. In particular, this research proposes and verifies a more nuanced, contingent model of abusive supervision in the domain of failure learning. Our results demonstrate that the impact of supervisor hostility is not uniformly destructive; rather, it exhibits a contingent valence shift. By showing that task variety can flip the direction of the indirect effect (from significantly negative to non-significantly positive), we challenge the one-size-fits-all view of leadership destructive effects. This contributes to the abusive supervision literature by identifying a structural boundary that determines when the dark side of leadership prevails and when it is potentially neutralized.

Second, we identify fear of failure as a novel affective mechanism that captures the primordial arousal-depletion tension of abuse, distinguishing it from existing rational-cognitive paths. Existing research on the double-edged effects of abusive supervision has primarily relied on attributional filters (Liao et al., 2021; Yu and Duffy, 2021) or achievement goal orientations (Wang et al., 2024; Jiang and Lin, 2025). These frameworks suggest that the divergence in behavior results from deliberate cognitive processing (e.g., attributing abuse to performance promotion vs. injury motivation) or stable personality traits (e.g., performance-approach orientation). In contrast, our fear of failure mechanism highlights a more immediate, pain-driven affective dynamic. Unlike positive attribution-driven reflectivity (Liao et al., 2021), self-blame emotion (Tröster and Van Quaquebeke, 2021), and achievement-oriented adaptation like work effort (Wang et al., 2024) or attentiveness (Srikanth et al., 2022), fear of failure represents a situational emotion that captures the “sharp sting” of abuse. It reveals a unique spillover effect in which personal hostility permeates the employee’s cognitive appraisal of the task itself. This fear emotion then increases or decreases the learning from failure depending on the extent of task variety. While the positive path via fear was non-significant in our study, documenting this shift is theoretically meaningful: it suggests that while fear has the motivational potential to drive preventative investment (Cheng and McCarthy, 2018), this affective arousal is fragile and operates as a resource buffer rather than a deliberate strategic choice. This distinguishes our findings from the rationalized adaptations found in prior studies, emphasizing that the environment (task variety) must first alleviate the depletion before the arousal can be channeled.

Thirdly, our research shifts the focus of contingent factors from individual differences to structural job characteristics in determining the double-edged outcomes of abusive supervision. Existing literature on the divergent effects of leader hostility has extensively cataloged various individual differences as the primary moderators that steer subordinates toward either constructive or destructive paths. For instance, research has established that subordinates’ goal orientations are decisive: while a high performance-approach orientation enables subordinates to channel the sting of abuse into increased effort, a performance-avoidance orientation exacerbates the threat and leads to withdrawal (Wang et al., 2024). Similarly, attributional styles -such as the tendency to perceive abuse as performance promotion versus injury initiation -act as decisive filters that determine whether a subordinate engages in functional adaptation or dysfunctional retaliation (Liao et al., 2021; Tröster and Van Quaquebeke, 2021). Other dispositional factors, such as an internal locus of control or strong ethical values, have likewise been identified as critical diverters (Kim et al., 2020; Islam et al., 2021).

While these studies emphasize who the employee is, we highlight that the direction of the double-edged effect is also dictated by what the employee does. By introducing task variety as a moderator, we demonstrate that the structural complexity of the work itself acts as a primary filter for leadership effects. Specifically, our findings show that high task variety serves as a vulnerability factor that exacerbates the negative, resource-depleting edge of the sword, leading to a significant collapse in learning. Conversely, low task variety acts as a structural buffer that neutralizes this toxicity.

Finally, we contribute to the debate on the mixed effects of negative emotions by identifying a resource-based boundary condition. Our findings reconcile the conflict between the interference view (Shepherd, 2003) and the functional view (George and Zhou, 2002). We clarify that the functional potential of negative affect is restricted by the resource price of the activity. By demonstrating that task variety determines whether fear facilitates detail-oriented focus or paralyzing frustration, we provide a clearer theoretical roadmap for predicting when the “sharp sting” of negative emotions will hinder or help organizational learning.

In sum, drawing on JD-R and COR theories, we argue that the functional edge of the sword is not merely a product of individual willpower or rationalization, but is strictly contingent upon a resource-demand fit. By documenting that the destructive path is significantly activated only when task demands are high, we provide a more balanced perspective on the contingency of abuse. Specifically, our results suggest that the environment acts as a critical filter: while high task variety amplifies the sting of abuse into a significant paralyzing blow to learning, low task variety serves as a structural buffer that neutralizes this toxicity, preventing a total collapse of learning efforts even if it does not fully catalyze them into a significant positive drive. This adds a critical structural dimension to the leadership literature, suggesting that job design is just as vital as personality in determining whether the “sharp sting” of abuse remains a debilitating drain or is rendered benign.

### 6.2 Practical implications

Beyond theoretical contributions, our findings offer actionable insights for organizations to manage the fallout of destructive leadership. To address the detrimental effects identified, we propose several specific instruments to cultivate job resources effectively.

Our study reveals that task variety is a vulnerability factor that amplifies the toxicity of abuse. Organizations should deploy a task-resource mapping tool – a diagnostic instrument that evaluates the variety and complexity of tasks against available cognitive resources. For employees in high-variety roles (e.g., R&D, creative design), where the resource price of failure learning is already steep, organizations should implement administrative load shedding (temporarily reducing non-essential variety) when interpersonal tension is high. By structurally simplifying tasks during periods of crisis, firms provide a resource buffer that prevents the significant collapse of learning activities.

Second, since abusive supervision spills over into a situational fear of failure, firms should move beyond abstract psychological safety and implement formal “post-failure learning kits”. These kits should include structured reflection templates that shift the focus from “Who is to blame?” to “What was the systemic trigger?”. By institutionalizing these debriefing sessions, the organization provides a cognitive scaffold that helps employees bypass the primordial fear response. For employees in low-variety tasks, where we found a fragile motivational potential for improvement, these instruments can act as the final catalyst needed to turn latent arousal into significant learning behaviors.

Third, to curtail abusive supervision at its source, organizations should replace generic performance reviews with resource-impact monitoring systems. These specific instruments track how supervisors affect their subordinates’ psychological capital (e.g., confidence, fear levels). When a supervisor’s resource-pulse score drops significantly – particularly in departments with high task variety – it should trigger an automatic leadership intervention protocol, which may include mandatory coaching or temporary reassignment. This ensures that the structural vulnerability of the work environment is matched with heightened organizational oversight.

Finally, given that fear of failure is a high-arousal, pain-driven emotion, organizations should offer cognitive-affective reappraisal training. This specific instrument teaches employees to de-link the “sharp sting” of interpersonal abuse from their appraisal of task failure. By equipping employees with the ability to quarantine the emotional spillover from a hostile leader, the organization helps them preserve the cognitive capacity necessary for analytical learning, even in a suboptimal interpersonal climate.

### 6.3 Limitations and future study

There are some limitations to this study. First, despite using a time-lagged design to measure variables, we still cannot establish causality among them. The nature of our research design requires caution regarding the conclusions we draw from the results. Longitudinal research or experimental studies could be implemented in the future. Second, we cannot entirely rule out common method bias (Podsakoff et al., 2012), particularly for the relationship between abusive supervision and fear of failure, because the two constructs were assessed from the same source (i.e., subordinates) at the same time. We encourage the use of rigorous field study designs that further rule out alternative explanations for the relationships we observed. Additionally, all measurements were self-reported by employees; future research could consider collecting data from both subordinates and supervisors, such as inviting supervisors to rate their followers’ learning from failure. Third, this study utilized a non-probability convenience sample, which may constrain the external validity of our findings. Future research could employ more diverse, probability-based sampling across different regions to further validate the generalizability of the asymmetrical double-edged sword model. At last, based on the conservation of resources theory, we proposed and tested the antecedent effect of abusive supervision on fear of failure, whereas previous empirical studies have also demonstrated a reverse relationship between negative emotions and abusive supervision (e.g., Pan and Lin, 2018; Eissa et al., 2020). Accordingly, the question may arise as to whether there is a reciprocal relationship between abusive supervision and fear of failure. Therefore, future scholars could test whether abusive supervision and fear of failure are mutually related.

## 7 Conclusion

In conclusion, this research demonstrates that the influence of abusive supervision on employees’ learning from failure is indirect and conditional. Drawing on the conservation of resources theory and the job demands-resources model, we show that abusive supervision elicits employees’ fear of failure, which subsequently influences their learning behavior contingent upon the level of task variety in their jobs. Our findings identify a critical boundary condition: under high task variety – where learning is most resource-intensive – abusive supervision suppresses learning from failure through fear of it. Conversely, under low task variety, the same fear motivates learning. In sum, this study examines abusive supervision in a failure experience context, and shifts from the rationalized view of abusive supervision to an affective, structural contingency framework within the context of failure learning.

## Data Availability

Publicly available datasets were analyzed in this study. This data can be found here: the datasets analyzed for this study can be found in the Harvard Dataverse, https://doi.org/10.7910/DVN/Y2MOLI.
