# Supplementary material for: Provoking or backfiring? A contingent model of how abusive supervision influences learning from failure through fear
Source: Front Psychol. 2026 Apr 22;17:1532064. doi: 10.3389/fpsyg.2026.1532064 (PMC13143604; doi:10.3389/fpsyg.2026.1532064)
Supplement: Supplementary file 1 [file Table_1.DOCX]

Supplementary Material

# APPENDIX

Table A Comparison of measurement models in the study Model

| Model | χ2/df | $\boldsymbol{\Delta}\boldsymbol{\chi}$^2^ | RMSEA | CFI | TLI |
| --- | --- | --- | --- | --- | --- |
| 1: Abusive supervision, fear of failure, task variety, learning from failure | 289.12/164 | - | 0.06 | 0.96 | 0.95 |
| 2: Combining abusive supervision and fear of failure | 861.98/167 | 572.87*** | 0.15 | 0.77 | 0.74 |
| 3: Combining abusive supervision and task variety | 1232.08/167 | 942.97*** | 0.18 | 0.65 | 0.60 |
| 4: Combining abusive supervision and learning from failure | 1102.07/168 | 812.95*** | 0.17 | 0.69 | 0.65 |
| 5: Combining fear of failure and task variety | 932.37/167 | 643.26*** | 0.16 | 0.75 | 0.71 |
| 6: Combining fear of failure and learning from failure | 959.26/168 | 670.15*** | 0.16 | 0.74 | 0.70 |
| 7: Combining task variety and learning from failure | 948.89/167 | 659.77*** | 0.16 | 0.74 | 0.71 |

**Notes.** *** *p <* 0.001.
